# Rearrangement of N-Terminal α-Helices of *Bacillus thuringiensis* Cry1Ab Toxin Essential for Oligomer Assembly and Toxicity

**DOI:** 10.3390/toxins12100647

**Published:** 2020-10-08

**Authors:** Sabino Pacheco, Jean Piere Jesus Quiliche, Isabel Gómez, Jorge Sánchez, Mario Soberón, Alejandra Bravo

**Affiliations:** Departamento de Microbiología Molecular, Instituto de Biotecnología, Universidad Nacional Autónoma de México (UNAM), Cuernavaca, Morelos 62250, Mexico; spacheco@ibt.unam.mx (S.P.); jean_19_2014@hotmail.com (J.P.J.Q.); isabelg@ibt.unam.mx (I.G.); jsanchez@ibt.unam.mx (J.S.); mario@ibt.unam.mx (M.S.)

**Keywords:** *Bacillus thuringiensis*, Cry1ab toxin, oligomer assembly, Föster Resonance Energy Transfer (FRET), disulfide bridges

## Abstract

Cry proteins produced by *Bacillus thuringiensis* are pore-forming toxins that disrupt the membrane integrity of insect midgut cells. The structure of such pore is unknown, but it has been shown that domain I is responsible for oligomerization, membrane insertion and pore formation activity. Specifically, it was proposed that some N-terminal α-helices are lost, leading to conformational changes that trigger oligomerization. We designed a series of mutants to further analyze the molecular rearrangements at the N-terminal region of Cry1Ab toxin that lead to oligomer assembly. For this purpose, we introduced Cys residues at specific positions within α-helices of domain I for their specific labeling with extrinsic fluorophores to perform Föster resonance energy transfer analysis to fluorescent labeled Lys residues located in Domains II–III, or for disulfide bridges formation to restrict mobility of conformational changes. Our data support that helix α-1 of domain I is cleaved out and swings away from the toxin core upon binding with *Manduca sexta* brush border membrane vesicles. That movement of helix α-2b is also required for the conformational changes involved in oligomerization. These observations are consistent with a model proposing that helices α-2b and α-3 form an extended helix α-3 necessary for oligomer assembly of Cry toxins.

## 1. Introduction

Pore-forming toxins (PFTs) are proteins produced by several pathogenic bacteria to disrupt the membrane integrity of their target cells, playing an important role in their virulence to invade or colonize their hosts. Most PFTs are produced as soluble proteins that must undergo substantial conformational changes to penetrate the hydrophobic barrier of the membrane bilayer. In different PFTs, oligomerization is a prerequisite for membrane insertion [1,2]. Oligomer assembly of PFTs requires structural rearrangements, allowing intermolecular interaction between monomers to form ring-shaped structures capable of making pores in the membrane [3,4,5]. The PFTs are classified in β- or α-PFT according to the secondary structure that is inserted into the membrane.

*Bacillus thuringiensis* (Bt) bacteria produce different PFTs highly lethal for invertebrates, mainly insects. During vegetative growth, different Bt strains secrete Vip toxins, and in synchrony with the sporulation phase synthetize Cyt and Cry toxins, accumulating them as parasporal crystals [6,7]. All of these toxins are PFTs, and some of them are used worldwide to control insect pests in agriculture or mosquitoes that transmit infectious diseases to humans. Among the Bt toxins described so far, Cry toxins are most abundant, and at least three different non-phylogenetically related Cry families have been found, such as the three-domain Cry toxins (named Cry), the Cry Bin-like (now named Tpp and Gpp) and the Cry Mtx-like toxins (now named Mpp and Mtx) [6,7,8]. 

The Cry family of toxins is considered as α-PFT since domain I, composed of seven α-helices, is involved in oligomerization and membrane insertion. These proteins are produced as protoxins with two possible sizes of 130 or 70 kDa. After ingestion by the target insect pest, N- and C-terminal regions are cleaved out by midgut proteases, releasing a 65 kDa soluble globular protease-resistant core composed of three domains. Domains II and III are composed of β-strands and are involved in binding to receptors located in the apical membrane of midgut epithelial cells of the insect larvae [9]. The interaction of Cry toxins with those receptors triggers the formation of oligomers whose three-dimensional structure remains unknown. It is proposed that the hairpin conformed by helices α4–α5 of domain I is implicated in membrane insertion to form the pore [10,11]. In the case of the Cry1A’s toxins it was shown that, after receptor interaction and protease activation, toxin complexes forming oligomers with an apparent molecular weight of 150–250 kDa generate electrical conductivity in black lipid bilayers, suggesting that oligomeric structures may be composed by 3–4 monomeric subunits [9]. The formation of a trimeric complex has been proposed for Cry1Ab and Cry4Ba oligomer formation observed by SEM and AFM in synthetic liposomes [12,13]. However, other reports support an arrangement of Cry1A toxins in tetrameric structures after liposome interaction [14].

For oligomer assembly, it was reported that after receptor binding additional proteolysis of helix α-1 at the N-terminal end region of Cry1Ab was required [15]. However, there are no reports describing the conformational changes in the N-terminal region of this protein leading to oligomer formation. For this reason, we decided to study the conformational changes at the N-terminal region of Cry1Ab by different methodologies to determine the conformational changes that occur during oligomerization of this protein. Interestingly, the monomeric structure of Cry4Ba and Cry5Ba toxins revealed by X-ray crystallography showed that the structural region corresponding to the helices α-1 and α-2a was cleaved out in a corresponding position located 50 residues upstream of the end of helix α-3, resulting in a trimeric structure organization of the diffracted Cry4Ba and Cry5Ba crystals (pdb: 1W99 and 4D8M), even though these two α-helices are present in the primary sequence of these proteins [8,16,17]. In these trimeric structures, the Cry4Ba and Cry5Ba proteins presented an extended helix α-3 comprising the loop region and helix α-2b. This long helix α-3 is located at the central core of the trimeric conformation presented by both proteins, showing multiple contacts with helices α-3, α-4 and α-6 from the adjacent monomers [16,17]. We previously showed that helix α-3 plays an important role in Cry toxin oligomerization, where intermolecular salt bridges were shown to participate in oligomerization and toxicity [18,19,20]. Furthermore, conserved charged residues found in the loop between helices α-2b and α-3 were shown to be involved in a salt bridge with adjacent monomers in several Cry toxins, implying that a conformational change of this loop into an α-helix is required to allow oligomer assembly [19]. In fact, the residues D129 and K131 of Cry5Ba toxin, located in this loop region, form an intermolecular salt bridge in the trimeric conformation of this protein, and mutations affecting these residues resulted in inactive toxins [19]. Similar evidence was observed by in silico modeling data of helix α-3 of domain I of Cry1A toxin [21]. Therefore, we hypothesized that a conformational change at helices α-2 and α-3 forming an extended helix α-3 is needed for oligomerization of Cry proteins.

To further analyze the conformational changes that occur in domain I leading to oligomer formation, in this work we designed a series of mutants to perform Föster resonance energy transfer (FRET) distance analysis between some α-helices of domain I and domains II–III of Cry1Ab toxin. We selected to work with Cry1Ab, since most of the work that has been done about the mechanism of action of Cry proteins has been done with this toxin. This protein also has an identical sequence to domain I of Cry1Aa, which has a known structure. We also constructed mutants for introducing disulfide bridge formation to restrict conformational changes. Cry1Ab activated toxin does not contain cysteine (Cys) residues, allowing the introduction of Cys residues at specific positions within helices α-1 and α-2b for specific labeling with extrinsic fluorophores or for the introduction of disulfide bridge formation. Our data support that helix α-1 of domain I is cleaved and swings away from the toxin upon binding with BBMV of *Manduca sexta*. We also show that a conformational change in helix α-2b is needed for oligomerization. These observations are consistent with the proposed hypothesis that helices α-2b and α-3 undergo a conformational change, forming an extended helix α-3 for oligomer assembly of Cry toxins necessary for their insecticidal activity.

## 2. Results

### 2.1. Prediction of Secondary Structure of the Hairpin between Helices α-2b and α-3 

**Figure 1:**
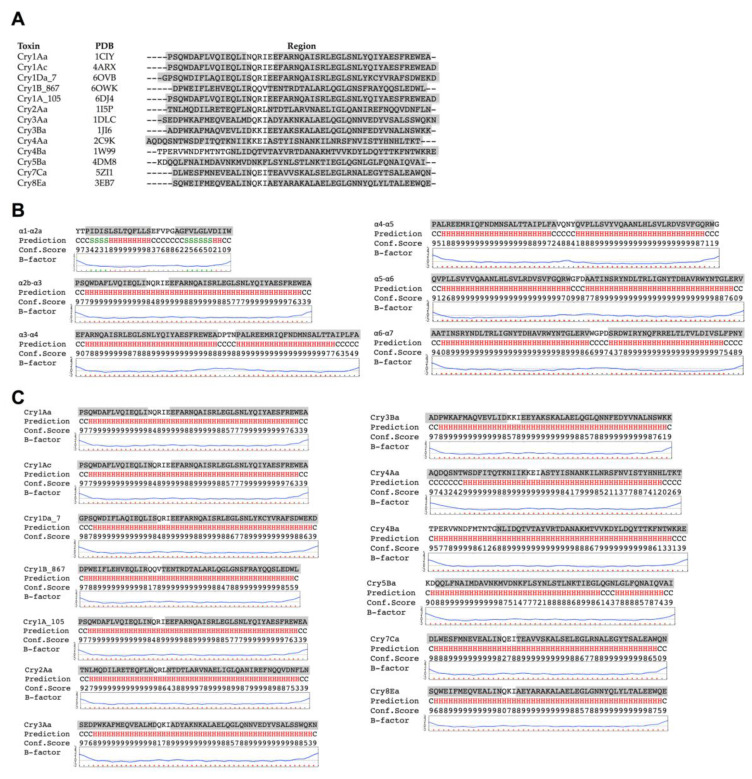
Analysis of the amino acid sequences of helices α-2b and α-3 of Cry1Ab. (**A**) Alignment of the amino acid sequence of regions containing helices α-2b and α-3 from Cry toxins, which have available structures. The α-helix structures observed in their three-dimensional structures are shown in gray. (**B**) Prediction of the secondary structure of the Cry1Ab hairpin regions among all α-helices of domain I, by using the i-TASSER server. H, α-helix; S, β-strand; C, coil (**C**) Prediction of the secondary structure of the coiled toxin regions between helices α-2b and α-3 of different Cry toxins by using the i-TASSER server, showing high probability to form an extended helix α-3. H, α-helix; S, β-strand; C, coil.

All three-dimensional structures resolved from monomeric Cry toxins contain seven α-helices, where the helix α-2 is bended, forming two small helices named α-2a and α-2b, which are connected by a flexible loop with helix α-3 (Figure 1A). The only exceptions are Cry4Ba and Cry5Ba toxin structures that contain a longer helix α-3 and lack helices α-1 and α-2a, although both helices α-1 and α-2 are present in their primary sequence (Figure 1A). We used the i-TASSER server to predict the secondary structure of the hairpin region between helices α-2b and α-3 from Cry1Ab (Figure 1B). The B-factor shown in this figure indicates the extent of the inherent thermal mobility of residues in the protein, where B-factor values higher than 0 indicate residues less stable in the analyzed structures. Based on the results from the i-TASSER prediction and B-factor values, we concluded that the region containing helices α-2b and α-3 from Cry1Ab potentially formed a single stable α-helix structure, while the rest of the loops between the other helices of domain I were predicted to remain as coils (Figure 1B). Similar analysis to predict the secondary structure of the hairpin region between helices α-2b and α-3 from other Cry toxins showed that in all of these toxins this region also could form a single stable α-helix structure.

### 2.2. Analysis of the Conformational Changes at the N-Terminal Region of Cry1Ab Toxin by FRET Assays

A structural model of the Cry1Ab was obtained based on the coordinates of the Cry1Aa structure (pdb:1CIY) as described in the methods section. These two proteins shared the same domain I amino acid sequence and structure. With the aim to analyze possible conformational changes at the N-terminal end of Cry1Ab domain I during its transition of monomer to oligomer conformation, we decided to use the FRET assays strategy. Activated Cry1Ab monomer lacks Cys residues and contains only three lysines (Lys), where two of them are located in domain III and one in domain II. Residues K403 and K490 are exposed to the solvent, while the third, K470, is buried within domain III. In order to perform FRET assays we constructed two mutants with a single Cys residue localized either in helix α-1 (Cry1Ab S41C) or in helix α-2b (Cry1Ab S71C) of domain I. The resulting mutant toxins showed similar insecticidal activity to Cry1Ab against *M. sexta* larvae, indicating that these mutants retained their binding ability with receptors and did not have major conformational constrains (Table 1). 

**Figure 2:**
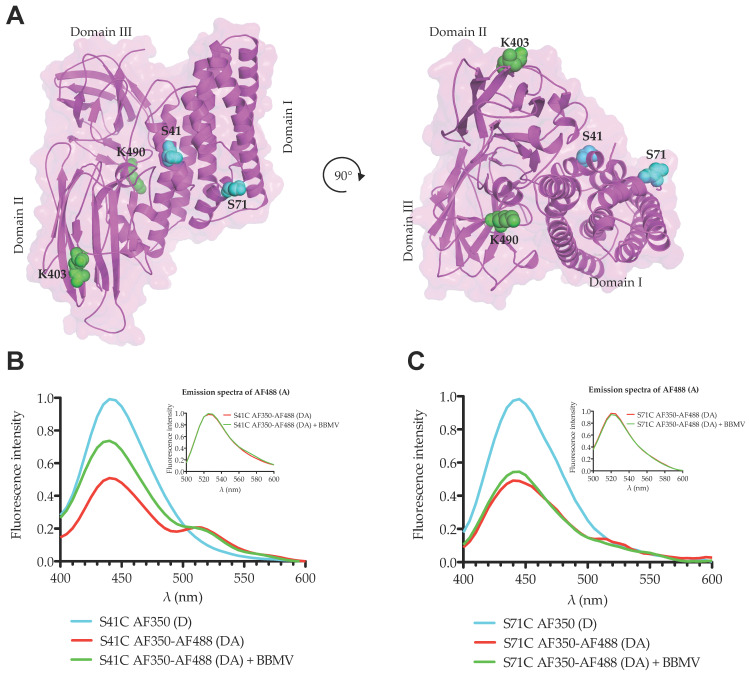
FRET analysis during oligomerization of Cry1Ab toxins. (**A**) Model structure of Cry1Ab toxin showing labeled residues. The introduced Cys residues S41C and S71C labeled with Alexa Fluor 350 _(D)_ are colored in cyan, and the exposed Lys residues K403 and K490 labeled with Alexa Fluor 488 _(A)_ are colored in green. (**B**) FRET assay with Cry1Ab S41C mutant. (**C**) FRET assay with Cry1Ab S71C mutant. Cyan lines show emission spectra of the donor fluorophore Alexa Fluor 350 _(D)_ bound to the introduced Cys residues in the mutant toxins in solution. Red lines show emission spectra of the D fluorophore bound to the Cys residues when these mutants were also labeled with acceptor fluorophore Alexa Fluor 488 _(A)_ bound to the Lys residues in solution _(DA)._ Green lines were similar proteins labeled with donor and acceptor fluorophores _(DA)_ analyzed after induction of oligomer formation by incubation with *M. sexta* BBMVs. Inserts show emission spectra of Alexa Fluor 488 _(A)_ of the double labeled mutants _(DA),_ which were excited with a wavelength of 488 nm to confirm that samples analyzed in the presence or in the absence of BBMVs had the same amount of the acceptor protein.

Cys-substituted residues S41C and S71C localized in helix α-1 or α-2b, respectively. These residues were labeled with the fluorescent dye Alexa Fluor 350 to act as donor (D), while intrinsic K403 and K490 were labeled with Alexa Fluor 488, acting as acceptor (A) in the same mutant toxin. According to the built structural model of Cry1Ab toxin, the average molecular distance between S41 or S71 with K403 and K490 was lower than 50 Å (Figure 2A), which corresponds to the Föster radius distance (*R*_0_) of the two selected fluorescent dyes (Alexa Fluor 350 as D, and Alexa Fluor 488 as A). At this distance, 50% of FRET energy transfer theoretically occurs. We first analyzed emission spectra of D fluorophore in the absence of A fluorophore of Cry1Ab S41C-D and Cry1Ab S71C-D mutants when these proteins were in the solution as monomeric structures (Figure 2B,C, cyan lines). FRET measurements were subsequently performed with the double labeled Alexa Fluor 350 and Alexa Fluor 488 (DA) Cry1Ab S41C-DA and Cry1Ab S71C-DA mutants in the same solution conditions. The fluorescence of D in the presence of A (DA) decreased approximately 50% of the total fluorescence emission observed in absence of A (Figure 2B,C, red lines), which is consistent with the molecular distance observed in the structural model of Cry1Ab protein. To determine whether helix α-1 is cleaved out or wrapped off upon oligomerization, the two mutants Cry1Ab S41C-DA and Cry1Ab S71C-DA were incubated with *M. sexta* brush border membrane vesicles (BBMVs) to induce their oligomerization. FRET analysis under such conditions showed that the emission of the D fluorophore in the presence of A of the double labeled mutant Cry1Ab S41C-DA was considerably higher (Figure 2B, green line), around 70% of the total fluorescence emission, when compared to the same mutant Cry1Ab S41C-DA in the solution (Figure 2B, red line), supporting that the loss of FRET energy transfer between D and A in the oligomeric structure could be due to the cleavage of helix α-1, which could have been lost or swung away from the toxin core. In contrast, when the double labeled mutant Cry1Ab S71C-DA was analyzed with BBMVs, we observed that the fluorescence emission of D fluorophore (Figure 2C, green line) was very similar, just slightly higher than in the solution (Figure 2C, red line), supporting that helix α-2b was not cleaved out upon toxin oligomerization, suggesting that only a subtle conformational change occurred in helix α-2b upon oligomerization.

### 2.3. Restriction of the Conformational Changes at the N-Terminal Region of Cry1Ab Toxin by Introducing Disulfide Bridges

**Figure 3:**
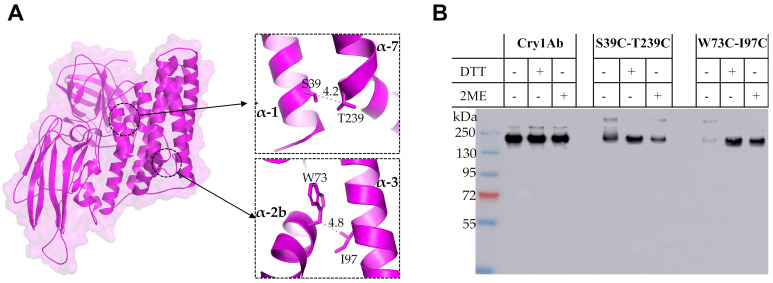
Mutagenesis to restrict mobility of Cry proteins by disulfide bridges. (**A**) Structure of Cry1Ab showing the distance between residues chosen for disulfide bridging. (**B**) Oligomerization assay of cross-linked mutants by disulfide bridges. Oligomer formation was analyzed by western blot using anti Cry1Ab-antibody after incubation with BBMVs isolated from *M. sexta* larvae. These assays were done in the absence and in the presence of reducing agents: DTT, dithiothreitol; or 2-ME, 2-mercaptoethanol. Pre-stained molecular weight markers PageRuler Plus (ThermoFisher Scientific, Waltham, MA, USA) are shown in the first lane.

To further analyze the conformational changes in the N-terminal region of Cry1Ab during oligomerization, intramolecular disulfide bridges were engineered among different helices of Cry1Ab toxin to restrict their flexibility during oligomer assembling. Typically, the optimal distance of the β-carbons of two amino acids forming a disulfide bond is approximately 5.5 Å [22]. Thus, to analyze if the removal of helix α-1 and the helix α-2b conformational change are required for oligomer assembly, we carefully selected two residues that were at the appropriate locations suitable for disulfide bridge formation between two Cys residues introduced by site directed mutagenesis. We selected to replace S39 located at helix α-1 and T239 at helix α-7 by Cys residues in the double mutant Cry1Ab S39C-T239C, since these residues have a distance of 4.2 Å between their β-carbons. In addition, W73 and I97 amino acids were replaced with Cys residues in the double mutant Cry1Ab W73C-I97C, since these amino acids located in helices α-2b and α-3 have a molecular distance of 4.8 Å between their β-carbons. As controls, we constructed all of the single Cys mutations of these selected residues (Figure 3A). The double mutants Cry1Ab S39C-T239C and Cry1Ab W73C-I97C produced similar parasporal crystal inclusions to Cry1Ab. The toxicity data against *M. sexta* larvae revealed that mutant Cry1Ab S39C-T239C showed a comparable toxicity to Cry1Ab (Table 1). In contrast, the Cry1Ab W73C-I97C mutant was severely affected in toxicity (>1104 folds higher LC_50_ value). We also analyzed the crystal production and toxicity of the control single Cys mutants. With the exception of the Cry1Ab T239C mutant, which was affected in its crystal production in the Bt transformant strain, all of the other single Cys mutants formed crystal inclusions in the Bt strain and showed similar insecticidal activity to Cry1Ab wild type (Table 1).

To determine if cross-linked Cry1Ab mutants by the introduced disulfide bridges are affected in their oligomer assembly, proteins were incubated with BBMVs isolated from *M. sexta* and oligomer formation was analyzed by western blot using anti-Cry1Ab antibody to recognize the high molecular band that corresponds to the oligomer structure. The buffers used for oligomerization and SDS-PAGE assays were prepared in the absence and in the presence of reducing agents to analyze the effect of the potential formation of disulfide bonds. Figure 3 shows that Cry1Ab and the Cry1Ab S39C-T239C mutant were able to form oligomer structures with or without reducing agents (Figure 3B). In contrast, the Cry1Ab W73C-I97C mutant was severely affected in the formation of oligomers in the absence of reducing agents, but the addition of dithiothreitol (DTT) or 2-mercaptoethanol (2-ME) during the incubation with BBMVs reconstituted the formation of oligomer structures (Figure 3B).

## 3. Discussion

The final structure of Cry toxin oligomers when inserted into the membrane remains unsolved. It was proposed that the interaction of Cry1Ab with membrane receptors triggers cleavage of helix α-1, facilitating the rearrangement of α-helices at the N-terminal end of domain I and leading to oligomer assembly [15,19]. Evidence based on disulfide cross-linking of various α-helices from domain I among them or between α-helices from domain I with domain II indicated that domain I swings out from domains II and III, resulting in insertion of helix α-5 into the membrane [10]. These studies showed that some cross-linked mutants resulted in the loss of pore formation activity in black lipid bilayers, which could be restored by reduction of the disulfide bonds, leading to the proposal that conformational changes involving several helices of domain I are required for pore formation [10]. In addition, protease protection analysis of Cry1Ac protein inserted into the membrane identified that only helix α-1 is highly susceptible to proteolysis, suggesting that this region is not inserted into the membrane [23]. Finally, studies using synthetic peptides corresponding to domain I α-helices indicated that helix α-1 peptide was the only one of the seven fluorescently labeled α-helices peptides that did not bind to phospholipid vesicles [24]. 

In this work, we analyzed the conformational changes of Cry1Ab upon oligomerization in the presence of BBMVs from *M. sexta*. We used two different strategies to perform these studies. First, we analyzed the conformational changes by FRET. FRET is an excellent assay to measure molecular distances between two fluorescent molecules that are located in a range of 20–100 Å when the emission spectrum of the donor molecule (D) overlaps with the excitation spectrum of the acceptor molecule (A). We selected two fluorescent dyes (Alexa Fluor 350 and Alexa Fluor 488) with a Förster distance (*R*_0_) value of 50 Å, indicating that if two residues labeled with these D and A fluorophores are separated by 50 Å, the reduction in fluorescence of the D molecule in the presence of the A molecule will be 50% due to their FRET interaction. We labeled two residues from domain I (one located in helix α-1 and the other in helix α-2b) with the donor Alexa Fluor 350 and performed FRET analysis on Lys residues located in domains II or III at approximately 50 Å distance. Our data showed that in solution the reduction in fluorescence of D fluorophore in the presence of the A molecule corresponded to the expected 50% reduction from both mutant toxins. When we performed the same FRET analysis in the presence of BBMVs, which induced oligomerization of the Cry1Ab toxin, we observed that fluorescence of the D bound to helix α-1 (Cry1Ab S41C) in the presence of the A bound to Lys residues was considerably higher in the oligomer conformation of this mutant when compared to the same protein in solution, supporting that the loss of FRET energy transfer between D and A in the oligomeric structure could be attributed to a conformational change that is compatible with the cleavage or the swinging away of helix α-1. It is important to mention that BBMVs were prepared in absence of protease inhibitors and thus the cleavage of helix α-1 may be catalyzed by BBMV-anchored proteases. This observation is consistent with the report that helix α-1 and part of helix α-2a of the Cry1Ab N-terminal end were cleaved out as indicated by the N-terminal sequence of the oligomeric structure [15]. Interestingly, we still observed some FRET in the presence of BBMVs, which could be explained if not all toxin molecules interacted with the membrane or only some molecules were cleaved, also if helix α-1 swings away from domains II–III but still remains associated to the toxin. However, oligomerization assays with BBMVs revealed that only oligomers were associated with BBMVs, supporting that after cleavage the helix α-1 may swing away from the core toxin and remain associated with the toxin. The exact position of helix α-1 remains to be demonstrated; in any case, our data support that an important conformational change in helix α-1 is produced during oligomerization.

In contrast, the analysis of the double labeled mutant Cry1Ab S71C-DA, where the donor is located in helix α-2b, in the presence of BBMVs, revealed that the fluorescence emission of D fluorophore was very similar to that observed in solution, just slightly higher, suggesting certain flexibility of helix α-2b, which is also consistent with our previous report where it was proposed that helices α-2b and α-3 are arranged to form a unique extended helix α-3 [19]. These results support that upon oligomerization helix α-1 has to be removed from the core toxin to allow the conformational change of helix α-2b, leading to an extended α-3 helix to establish contacts with adjacent monomers.

The second strategy used to analyze the conformational changes of Cry1Ab toxin during its oligomerization was the introduction of two Cys residues to allow disulfide bridge formation that could restrict secondary structure mobility; thus, the separation of two adjacent α-helices is expected to be limited by the introduced cross-linking due to disulfide bridge formation. Interestingly, we found that a single Cry1Ab T239C mutant was affected in its crystal production in Bt and thus its toxicity was not analyzed, but the double Cry1Ab S39C-T239C mutant produced crystals that were toxic to *M. sexta*, indicating that the disulfide bridge stabilized the double mutant protein, allowing its accumulation in crystal inclusions. These data also indicated that the cross-linking between helices α-1 and α-7 did not affect the conformational changes required for oligomer formation.

The single mutants Cry1Ab W73C and Cry1Ab I97C were able to produce crystal inclusions in Bt and were toxic to the larvae, similar to the wild type Cry1Ab toxin. In contrast, when helix α-2b was linked with helix α-3 in the double Cry1Ab W73C-I97C mutant, this protein was severely affected in toxicity against *M. sexta* and also in its capacity to form oligomer structures, which were observed only in the presence of reducing agents, DTT or 2-ME, during its incubation with BBMVs. These data suggest that a conformational change of helix α-2b is required for oligomer formation, and restriction of its mobility severely affects oligomerization and insecticidal activity. This result also shows that disulfide bridges remain stable in vivo since the toxicity of this double Cry1Ab W73C-I97C mutant was severely affected, regardless of the aforementioned reducing environment of lepidopteran larval gut [9]. It is possible that the introduced disulfide bridge is protected by the toxin structure or that the concentration of reducing agents in the midgut is not high enough to affect this particular bridge. In contrast, when we linked helix α-1 with helix α-7 in the Cry1Ab S39C-T239C mutant, this mutant was still able to form oligomer structures without reducing agents and retained toxicity to *M. sexta* larvae. These results suggest that helix α-1 remained attached to the toxin but did not impair the conformational change of helix α-2b upon oligomerization, probably because it is cleaved, allowing the helix α-2b conformational change to occur even though helix α-1 remains attached to the toxin by the disulfide bridge. Our FRET analysis suggested that helix α-1 may be cleaved out from the toxin upon oligomerization, but remains attached to the toxin, which is compatible with these results obtained with the restriction of helix α-1 mobility by disulfide bridge, showing that cross-linking helix α-1 with helix α-7 does not affect oligomerization. 

**Figure 4:**
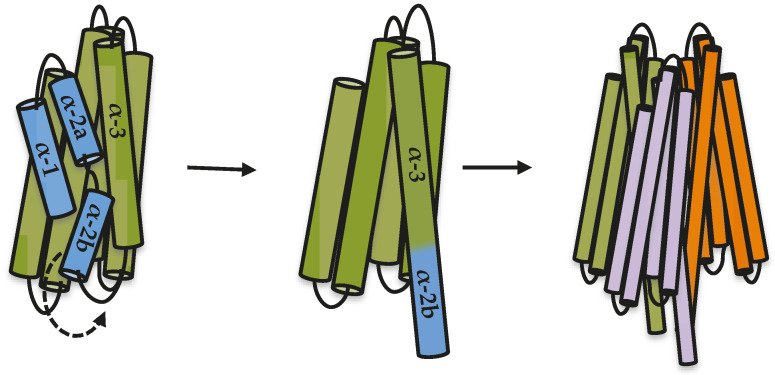
Model of the structural changes of domain I α-helices during Cry toxin oligomerization. We propose that a cleavage before helix α-2b is needed to induce oligomerization. This helix α-2b could then move freely and change its location to form an extended helix α-3 that is required to make further contacts with adjacent monomers during oligomerization. Additional changes must occur, such as the movement of helices α-4 and α-5, as has been proposed by previous works reported in the literature.

In silico prediction suggested that the hairpin between helices α-2b and α-3 h in Cry1Ab toxin is likely to have α-helix structure. A similar observation was previously shown when this region was analyzed for its capacity to form coiled coil structures by using a program designed to identify regions prone to form such coiled coils among putative α-helices [18]. The analysis by the coiled coils prediction program showed that this region was the only one within the Cry toxin core that has high potential to form such structures [18]. These two analyses performed with different algorithms support that this region has the flexibility and biochemical characteristics required for the conformational change leading to an extended α-3 helix formation required for facilitating oligomer formation. In agreement with these data, we previously showed that at least two conserved salt bridges may be formed between the predicted extended helices α-3 from adjacent monomers in the Cry family during oligomerization. The most conserved salt bridge in the Cry family is the one located in the loop region between helix α-2b of one monomer and helix α-3 from the adjacent monomer [19]. It is important to mention that such a salt bridge could only be formed if helix α-2b and the loop between this helix and helix α-3 changed their conformation, forming an extended helix α-3. In the case of Cry5Ba, mutations of the salt bridge located in the loop region between helix α-2b and helix α-3 affected toxicity to nematodes, supporting the functionality of this salt bridge for toxicity [19]. The Cry1Ab does not contain this salt bridge but has another salt bridge formed by two residues in helix α-3 from adjacent monomers, which has been shown to be essential for toxicity since mutations in that salt bridge (Cry1Ab R99E or Cry1Ab E101K) severely affected oligomerization and toxicity, while reversed charged mutagenesis in the double mutant Cry1Ab R99E-E101R recovered both oligomerization and toxicity [19]. Interestingly, the introduction of the salt bridge located in the loop between helices α-2b and α-3 to a Cry1Ab mutant lacking the other nearby salt bridge (Cry1Ab R99E-N85D-R87K) also recovered toxicity and oligomer formation, supporting that this loop region must be structured as α-helix for oligomer assembly and toxicity [19]. Based on all results presented here and the previously published data, we propose a model to explain the first steps in oligomerization of Cry toxins, where the proteolytic cleavage between helix α-2a and helix α-3 releases helix α-2b, allowing it to move and rearrange to make an extended helix α-3 that is needed for oligomer formation (Figure 4). It has been proposed that additional changes must occur during insertion of a Cry oligomer into the membrane, such as the movement of helices α-4 and α-5 to finally be inserted into the membrane [10,11,12].

Overall our results show that oligomerization is a key step in the toxicity pathway of Cry1Ab toxin that is triggered by conformational changes of the N-terminal domain I, allowing the formation of a pre-pore structure capable of membrane insertion and pore formation.

## 4. Materials and Methods 

### 4.1. Site Directed Mutagenesis

Site directed mutagenesis was used to introduce selected mutations in the *cry1Ab* gene. Mutagenic PCR reactions were performed by using 200 ng of pHT315-Cry1Ab plasmid [25], 50 pmol of mutagenic primer and 20 μL of Phusion High-Fidelity PCR 2X Master Mix (ThermoFisher Scientific, Waltham, MA, USA). Thermocycling conditions were: one incubation at 98 °C for 1 min; 30 cycles of 98 °C for 10 s, 55 °C for 30 s and 72 °C for 5 min; and one incubation at 72 °C for 5 min. The PCR reaction was purified and methylated parental DNA was digested with 1 U of DpnI (New England Biolabs, Ipswich, MA, USA) for 2 h at 37 °C. The reaction was then electrotransformed in *Escherichia coli* DH5α and grown on an LB medium containing 100 μg/mL of ampicillin for 16 h at 37 °C. Plasmids were extracted and prepared for Sanger sequencing. Double mutants of Cry1Ab toxin were generated using the single mutants as the template for a second PCR mutagenic reaction. Primers used for mutagenesis were: S39C, 5′-CCC CAA TCG ATA TTT GCT TGT CGC TAA CGC-3′; S41C, 5′-CGA TAT TTC CTT GTG CCT AAC GCA ATT TCT TTT G-3′; S71C, 5′-GAA TTT TTG GTC CCT GTC AAT GGG ACG CAT TTC-3′; W73C, 5′-GGT CCC TCT CAA TGC GAC GCA TTT CTT GTA C-3′; I97C, 5′-GGA ACC AAG CCT GTT CTA GAT TAG AAG G-3′; T239C, 5′-GAG AAT TAA CAC TAT GTG TAT TAG ATA TCG-3′.

### 4.2. Production and Purification of Cry1Ab Toxin

All plasmids were transformed in *E. coli* SCS110 by thermal shock to obtain non-methylated plasmids and then electrotransformed into the acrystalliferous Bt 407 strain, as previously described [26]. The Bt transformant strains were grown on an HCT medium [27] supplemented with 10 μg/mL of erythromycin for 72 h at 30 °C. A spore/crystal mixture was harvested and washed three times with wash solution (0.3 M of NaCl, 10 mM of EDTA), followed by three washes with 1 mM of PMSF (final concentration). Cry1Ab crystals were solubilized during 1 h with an alkaline buffer (0.1 M of NaHCO_3_, 0.02% 2-mercaptoethanol, pH of 10.5), and then neutralized with 1 M of Tris-HCl with a pH of 8.0 and activated with trypsin at a 1:20 ratio (*p/p*) for 1 h at 37 °C. Activated Cry1Ab toxin was purified by ion exchange with a HiTrap Q HP column (GE Healthcare, Little Chalfont, UK) connected to an AKTA FPLC system (GE Amersham Biosciences, Little Chalfont, UK). Protein concentration was estimated by a Bradford assay using a standard curve of BSA.

### 4.3. Insect Bioassays

Twenty-four well plates were filled with an artificial diet for lepidopteran insects [28] and the surface was contaminated with different concentrations of Cry1Ab toxins (1.25 to 20 ng/cm^2^) in triplicate. The maximum dose analyzed for Cry1Ab W73C-I97C was 2000 ng/cm^2^. A total of 24 neonate larvae of *M. sexta* per plate were reared at 28 °C, with 65% relative humidity and a 16/8 h dark–light photoperiod. Mortality was registered after seven days and 50% lethal concentration (LC_50_) was estimated using a Probit analysis with POLO Plus LeOra software. A negative control without a toxin addition was included in the bioassay. 

### 4.4. Preparation of BBMVs

*M. sexta* larvae from third instar reared at the laboratory were used to prepare brush border membrane vesicles (BBMVs) by the differential precipitation method with MgCl2 as reported before [29]. The total protein amount of the BBMVs was estimated by the Lowry assay using a standard curve of BSA and stored at −70 °C. Enrichment of BBMVs was calculated as previously reported [30], showing that aminopeptidase activity was five-fold higher in the BBMVs than the initial homogenate.

### 4.5. Oligomerization of Cry1Ab Toxin

Cry1Ab oligomers were produced by incubation of 1 μg of purified toxin with 10 μg of BBMV protein in 1 M of buffer NaHCO_3_ with a pH of 10.5 during 1 h at 30 °C. After incubation of Cry1Ab toxin with BBMVs, samples were ultra-centrifuged for 30 min at 55,000 rpm, 4 °C and the membrane pellet was recovered for further analysis. Oligomerization reactions were performed in presence or absence of reducing agents (0.02% 2-ME or 5 mM DTT). The samples were suspended in Laemmli loading buffer without 2-ME, heated at 50 °C for 3 min, separated by SDS-PAGE and detected by western blot using polyclonal anti-Cry1Ab and secondary goat anti-rabbit IgG-HRP (Santa Cruz Biotechnology, Dallas, TX, USA). Finally, oligomers were visualized with Luminol reagent (Santa Cruz Biotechnology, Dallas, TX, USA) in Amersham Imager 600 device (GE LifeScience, Little Chalfont, UK).

### 4.6. Labeling of Cry1Ab Toxin with Fluorescent Dyes

Purified Cry1Ab mutants were incubated with 3 mM of DTT and 1 mM of EDTA for 15 min at room temperature to improve the labeling with Alexa Fluor 350 C5-Maleimide (ThermoFisher Scientific, Waltham, MA, USA). The DTT was removed by size exclusion using Bio-Gel P-6 beads (Bio-Rad, Hercules, CA, USA), and proteins were quantified on a Nanodrop 2000 (ThermoFisher Scientific, Waltham, MA, USA) measuring the absorbance at 280 nm with a molar extinction coefficient of 85,260 M^−1^ cm^−1^. Proteins (200–300 μg) were then incubated with 20-fold molar excess of the probe in PBS with a pH of 7.2 during 2 h in darkness. The unbound label was removed by dialyzing exhaustively overnight against PBS with a pH of 7.2 at 4 °C. A fraction of Cry1Ab toxin labeled with Alexa Fluor 350 was labeled with the second dye Alexa Fluor 488 TFP ester (ThermoFisher Scientific, Waltham, MA, USA ). Unbound dye was removed by dialysis in 0.1 M of alkaline buffer NaHCO_3_ with a pH of 8.0 overnight at 4 °C. Quantification of toxin: dye conjugation was determined as described in the protocol TR0031 from ThermoFisher Scientific, Waltham, MA, USA. Labeled proteins were analyzed on SDS-PAGE and visualized by excitation of the SDS-PAGE gel with a UV light transilluminator and analyzed in an Amersham Imager 600 (GE LifeScience, Little Chalfont, UK).

### 4.7. FRET Measurements

Cry1Ab and mutant activated toxins in solution were prepared at 20 ng of protein/μL in 0.1 M of alkaline buffer NaHCO_3_ with a pH of 10.5. Donor sample “D” was labeled with Alexa Fluor 350, and donor acceptor sample “DA” was double labeled with Alexa Fluor 350 and Alexa Fluor 488. Oligomerization of Cry1Ab toxins was induced as described above. Fluorescence intensity was recorded in the spectrofluorimeter Infinite M1000 Pro (Tecan, Männedorf, Switzerland). The emission spectra were recorded at 25 °C from 400 to 600 nm with 5 nm increments, using an excitation wavelength of 350 nm in triplicate with D or with DA labeled mutants. The DA labeled mutants were also excited at 488 nm wavelength to analyze emission of A when the protein is in solution and in samples incubated with *M. sexta* BBMVs, in order to confirm that the same concentration of protein was used in both assays. Buffer and buffer plus BBMV spectra were subtracted and data were normalized. 

### 4.8. Prediction of Cry1Ab Structure

The three-dimensional structure of Cry1Ab toxin was modeled by homology on the SWISS-MODEL server (https://swissmodel.expasy.org), using as a template the X-ray structure of Cry1Aa (PDB code 1CIY) [31]. Figures were prepared with PyMol software. The secondary structure of the hairpin regions that corresponds to all α-helices of domain I of Cry1Ab was predicted on the i-TASSER server (https://zhanglab.ccmb.med.umich.edu/I-TASSER) [32].

## Figures and Tables

**Table 1:** Toxicity of Cry1Ab toxins against *Manduca sexta* larvae.

Toxin	LC_50_ in ng/cm^2^	Localization of Mutation
	(Fiducial Limits) *	
Cry1Ab	1.81 (1.3–2.5)	
Cry1Ab S41C	1.84 (0.1–3.3)	Helix α-1
Cry1Ab S71C	2.5 (1.2–3.1)	Helix α-2b
Cry1Ab S39C-T239C	0.86 (0.6–1.1)	Helices α-1 and α-7
Cry1Ab W73C-I97C	>2000	Helices α-2b and α-3
Cry1Ab S39C	0.93 (0.6–1.2)	Helix α-1
Cry1Ab T239C	ND	Helix α-7
Cry1Ab W73C	1.4 (0.2–3.4)	Helix α-2b
Cry1Ab I97C	1.5 (1.2–2.6)	Helix α-3

* Values of 95% fiducial limits are shown in parenthesis. ND: Not determined.
